# E2F5 status significantly improves malignancy diagnosis of epithelial ovarian cancer

**DOI:** 10.1186/1471-2407-10-64

**Published:** 2010-02-24

**Authors:** Narasimhan Kothandaraman, Vladimir B Bajic, Pang NK Brendan, Chan Y Huak, Peh B Keow, Khalil Razvi, Manuel Salto-Tellez, Mahesh Choolani

**Affiliations:** 1 Diagnostic Biomarker Discovery Laboratory, Department of Obstetrics and Gynaecology, Yong Loo Lin School of Medicine, National University Health System, 5 Lower Kent Ridge Road, 119074, Singapore; 2 Computational Bioscience Research Center (CBRC), 4700 King Abdullah University of Science and Technology (KAUST), Thuwal 23955-6900, Kingdom of Saudi Arabia; 3 Department of Pathology, National University Health System, Yong Loo Lin School of Medicine, 119074, Singapore; 4 National University Medical Institutes, Yong Loo Lin School of Medicine, National University Health System, 119074 Singapore; 5 Southend Hospital NHS Trust, Westcliff-on-Sea, Essex, UK; 6 Basildon & Thurrock University Hospital NHS, Foundation Trust, Basildon, Essex, UK

## Abstract

**Background:**

Ovarian epithelial cancer (OEC) usually presents in the later stages of the disease. Factors, especially those associated with cell-cycle genes, affecting the genesis and tumour progression for ovarian cancer are largely unknown. We hypothesized that over-expressed transcription factors (TFs), as well as those that are driving the expression of the OEC over-expressed genes, could be the key for OEC genesis and potentially useful tissue and serum markers for malignancy associated with OEC.

**Methods:**

Using a combination of computational (selection of candidate TF markers and malignancy prediction) and experimental approaches (tissue microarray and western blotting on patient samples) we identified and evaluated E2F5 transcription factor involved in cell proliferation, as a promising candidate regulatory target in early stage disease. Our hypothesis was supported by our tissue array experiments that showed E2F5 expression only in OEC samples but not in normal and benign tissues, and by significantly positively biased expression in serum samples done using western blotting studies.

**Results:**

Analysis of clinical cases shows that of the E2F5 status is characteristic for a different population group than one covered by CA125, a conventional OEC biomarker. E2F5 used in different combinations with CA125 for distinguishing malignant cyst from benign cyst shows that the presence of CA125 or E2F5 increases sensitivity of OEC detection to 97.9% (an increase from 87.5% if only CA125 is used) and, more importantly, the presence of both CA125 and E2F5 increases specificity of OEC to 72.5% (an increase from 55% if only CA125 is used). This significantly improved accuracy suggests possibility of an improved diagnostics of OEC. Furthermore, detection of malignancy status in 86 cases (38 benign, 48 early and late OEC) shows that the use of E2F5 status in combination with other clinical characteristics allows for an improved detection of malignant cases with sensitivity, specificity, F-measure and accuracy of 97.92%, 97.37%, 97.92% and 97.67%, respectively.

**Conclusions:**

Overall, our findings, in addition to opening a realistic possibility for improved OEC diagnosis, provide an indirect evidence that a cell-cycle regulatory protein E2F5 might play a significant role in OEC pathogenesis.

## Background

Ovarian epithelial cancer (OEC) remains the most lethal gynecological malignancy in Western countries [1,2]. Poor prognosis is due to the late stage at first presentation, and advances in surgery and chemotherapy have had small impact on survival. In contrast, patients who present with early-stage disease have a five-year survival of up to 95% after surgery alone, and may even be spared the toxic side effects of postoperative adjuvant chemotherapy [3]. Early detection of this lethal disease remains the most promising approach to improve the long-term survival and quality of life of patients with OEC [4]. Serum CA125 is a good tumor marker for monitoring patients with ovarian cancer after they have been appropriately treated, but is a poor biomarker for screening and detection of early OEC [5]. Screening strategies are being explored for the early detection of epithelial ovarian cancer, but these appear to still have limitations in their detection and high false positive rates [6,7]. Although susceptibility genes, such as BRCA1 and BRCA2, have been identified, a majority of ovarian cancers occur sporadically without known risk factors [8].

Over the past few years several groups have reported potential of gene expression profiling based on microarrays to study the expression patterns of different genes during onset of ovarian cancer [9,10]. Some of these markers identified using this technique are: (1) prostasin a serum marker for ovarian cancer [11]; (2) Mesothelin (MSLN) [12,13], (3) WFDC2 (HE4) a glycoprotein [14] (4) osteopontin [15]; (5) Bikunin [16]; (6) mammaglobin-2 (MGB2) [17]; (7) discoidin domain receptor 1 (DDR1) [18]; (8) claudin 3 (CLDN3) [18]; (9) epithelial cell adhesion molecule (Ep-CAM) [18]; and (10) E-cadherin [18]. These markers were studied individually and not examined as part of the whole process of oncogenesis that would provide compelling evidence of their role in the disease process, and their utility as potential OEC biomarkers.

There is vast amount of gene expression profiling data available in the public domain, as well as in various private databases for ovarian cancer [9]. More recently, focused efforts were made to exploit these vast valuable resources to identify potential markers for various cancers [19,20]. These strategies save precious time and avoid unnecessary duplication of experiments and, moreover, can help focus on the most promising experiments.

Cell-cycle genes and associated regulatory factors which play a key role in cancers are a key target for most of biomarker discovery efforts [21-23]. Cell-cycle machinery controls cell proliferation, and cancer is a manifestation of disrupted cell proliferation. Different phases in cell-cycle (G1, G1-S, S, and G2-M), show activities of several oncogenes and tumor suppressor genes that display a range of abnormalities with potential usefulness as markers of genesis and as prognostic markers for ovarian cancer [22]. Transcription factors (TFs) control the expression of various genes during these different phases of cell-cycle.

Previous reports have focused mostly on secretory proteins as markers of OEC. However, most of such proteins were ineffective in diagnosing the disease early. More recently, the use of TFs as markers for the disease itself have been reported and they have been detected in the blood [24-26]. In our study, the strategy was to investigate the regulatory mechanism of genes implicated by expression data as specific to OC and in this way to identify the key TFs associated with this malignancy which could aid in discriminating malignant condition from benign and healthy conditions. We hypothesized that over-expressed TFs, as well as those that are driving the expression of the OEC over-expressed genes, could be the key for OEC genesis and potentially useful tissue and serum markers for malignancy associated with OEC. The identified TFs in this study participate in a common control mechanism of several key over-expressed genes, such as keratins which are themselves involved in the cell structural integrity. The molecular processes during OEC might ultimately result in the release of oncoproteins into blood stream, particularly if implicated TFs are involved in the regulation of genes responsible for structural properties of extracellular matrix as are keratin encoding genes, or if they are themselves controlled in the similar fashion as such genes. The results of our bioinformatics analysis to determine relevant TFs guided by the gene expression results, is followed up by extensive experiments based on clinically relevant cases and revealed among the identified TFs, E2F5 that demonstrates a strong potential to improve diagnostics of OEC.

## Methods

### Patient serum samples

A total of 88 women, aged between 20 and 72 years, 40 benign, and 48 malignant cases (16 early, and 32 late cases) (Table 1) were recruited for open surgical or laparoscopic treatment at the Department of Obstetrics and Gynecology, National University Hospital, Singapore), from 1998 to 2006. The histologic type of ovarian cancer were classified as defined by International Federation of Gynecology and Obstetrics [27] staging [27] [FIGO Cancer Committee (1986).]. A total of 56 normal volunteers were recruited as controls that were diagnosed to have no cyst or any other type of malignancy. Venous blood from case and control groups (normal volunteers) were collected in 8-ml Vacuette serum tubes containing clot activating factor (Greiner Bio-One, Kremsmuenster, Austria). Tissue samples from cysts of ovarian carcinomas and benign ovarian cysts were collected during surgery without intraoperative spillage, and the histology confirmed by a pathologist. All samples were transported on ice to the laboratory, centrifuged immediately at 4°C for 10 minutes at 1500 g, and stored at -80°C until analysis. All samples were collected with informed consent process which was cleared by the Ethical approval from the National Healthcare Group Domain Specific Review Board (DSRB, Singapore).

**Table 1:** Clinical characteristics and age distribution of study samples (serum samples) used for E2F5 and CA125 expression studies.

	Number of cases	Age	Cancer subtypes				
			
			Serous carcinoma	Mucinous carcinoma	Endometroid carcinoma	Clear cell carcinoma	Others*
Normal	56	45.00 ± 15.00					
Benign	40	42.80 ± 16.65	5	11	14	0	10
Stage I/II	16	50.94 ± 12.16	2	4	6	3	1
Stage III/IV	32	52.32 ± 10.11	19	1	9	3	0

#### Computational selection of candidate TFs markers

We examined a representative set of OEC microarray expression data [28], which implicates 19 genes that were at least 5-fold over-expressed. Among these 19 genes, several were encoding for TFs and three genes were from the keratin group. Since keratins are involved in the cell integrity, we were interested to find if the over-expressed TF encoding genes are subjected to the same control mechanism as keratins or if the corresponding TFs are involved in the control of keratin genes. This would suggest a possibility that an over-expressed TF encoding genes may result in overproduction of the corresponding TFs that may leak out of cell in case of cell damage, making them available in body fluids, such as blood. Through analysis of promoter content of these 19 genes, we found genes that share a common promoter model and thus share common promoter elements (PEs). By a PE we consider a TF binding site (TFBS) and the DNA strand where TFBS is found. For example, in our notation "AREB6/-1" represents a PE that involves AREB6 binding site found on the reverse complement DNA strand. Transcription start sites (TSSs) of the analyzed promoters were determined using the same procedure as described by [29]. The procedure resulted in 10,255 highly accurate human promoters and we considered a region of [-800, +200] relative to the experimentally confirmed TSSs. Promoters' genomic sequences were extracted from the human genome release hg17 from http://hgdownload.cse.ucsc.edu/downloads.html#human. We used all available mammalian matrix models for TFBSs contained in TRANSFAC Professional database ver. 7.4, and mapped them to the promoter sequences. The *minSUM *profiles were used for threshold of the matrix models since these are based on the optimization that provides the minimum sum of false positive and false negative predictions of putative TFBSs. We compared promoter content of 19 overexpressed genes against the remaining human promoters, and calculated the overrepresentation index (ORI) based on the method introduced by [29,30]. The method has been successfully applied in several studies [29,31] and others. All TFBSs mapped to promoters were ranked according to decreasing ORI values. We analyzed content of promoters of 19 over-expressed genes based on mapped TFBSs that had ORI not less than 1.5. Based on that analysis we found the promoter model characteristic of the keratin gene group and respective PEs. The promoter model was characterized by the presence of AREB6/-1 PE, and one or more of the following three PEs: GBF/-1, Kr/+1 and XPF-1/-1. Additional six genes (E2F5, PAX8, ELF3, WFDC2 (HE4), MUC1, LCN2) from the 19 analyzed shared this promoter model, while the other 10 were void of such combinations. Thus, three genes encoding for TFs, E2F5, PAX8 and ELF3 were subjected to the same putative control model and also were found over-expressed in OEC. Consequently, we found four candidate TFs (AREB6, E2F5, PAX8, ELF3) that have a potential to play a role in OEC. More details on the modalities followed for analyzing over expressed genes in ovarian cancer and potential utility of transcription factors as therapeutic application is described in Additional file 1.

#### Malignancy prediction

We analyzed how well malignant cases can be predicted using Risk Malignancy Index (RMI), other clinical indicators and E2F5 status. In total, we considered 38 benign cases, and 48 malignant cases (16 early and 32 late). We had 13 features associated with each of the cases. The thirteen features we used include (1) age (2) stage of ovarian cancer at the time of detection (3) serum CA125 levels (4) size(cm) (5) ascites (6) metastases (7) presence of multilocular cyst (8) solid area (9) bilateral (10) ultrasound score (11) menopause score (12) RMI and (13) E2F5 status. Due to relatively small number of cases we first generated a set of 602 artificial cases based on the set of 86 benign and malignant ones, using the so-called The Synthetic Minority Oversampling Technique (SMOTE) algorithm [32]. We then applied the Kstar algorithm [33] to train the machine learning system to distinguish between malignant and benign cases using the artificial set of 602 cases. Then we applied the trained system to the original 86 cases. We conducted three experiments. In the first one we used all 13 features. In the second one we excluded from the original 13 features CA125 information. In the third one we excluded from the original 13 features the E2F5 status.

The classification performance was expressed using the accuracy and F-measure (for other measures of prediction quality see [34]). In what follows tp, tn, fp, fn stand for true positive, true negative, false positive, and false negative predictions, respectively. Accuracy is defined as:

while F-measure is defined as

where sensitivity and ppv (positive predictive value) are

#### Tissue microarray studies (TMA) studies

A total of 135 formalin-fixed, paraffin-embedded ovarian tissue samples (111 (43 + 68) benign and malignant tumors and 24 normal) from the National University Hospital of Singapore were arrayed as previously described [35]. These cases represented a cross-section of normal/physiological ovarian tissue-types, benign neoplasms and ovarian malignancies (see Table 2 for details). Briefly, hematoxylin and eosin-stained sections were reviewed to annotate representative tumor and normal areas. These were sampled with a 1.0-mm diameter tissue cylinder for normals and benign tumors and a 2.0-mm diameter tissue cylinder for borderline and malignant tumors and deposited into a "recipient" block using a tissue arraying instrument (ATA100, CHEMICON International Inc, Temecula, CA). Of the 68 malignant tumours, 7 serous tumours, 4 endometroid tumours, 2 clear cell tumours, 2 high grade adenocarcinomas not further characterized and 3 mixed epithelial tumours were arrayed in double punches due to bilateral ovarian involvement and to account for tumour heterogeneity. After TMA construction, 4-μm sections were prepared for hematoxylin and eosin staining verification of the accuracy of the TMA construction, and for E2F5 antibody analysis. This approach, in our hands, ensures a reliable representation of the biology of the tumour, when compared with full section analysis, and has been tested before in the confirmation of other novel biomarkers [36]. Immunohistochemistry was performed using the polyclonal antibody to the E2F5 transcription factor (ACRIS Antibodies GmbH, Germany), optimized in our laboratory for immunohistochemistry on full sections and TMA slides of formalin fixed, paraffin embedded tissues with a protocol that includes microwaving for heat induced epitope retrieval in DAKO Target Retrieval Solution at pH9 for 20 minutes. Our positive control cases used for the optimization of the antibody, comprising normal epidermis, showed expected staining for E2F5 in the basal and granular layers [37]. Our negative control was a sample from normal ovary. Not only was the sample negative throughout, there was also no background staining. The expression of E2F5 antibody was found to be cytoplasmic. While the expression for the antibody was not very strong, there was indeed a significant difference from normal and benign ovarian tissue. The results were independently scored by an experienced observer (BP) and confirmed by a second one (MST). The scoring system suggested a positive result if there was any detectable cytoplasmic staining in the lesional cells of interest as can be seen from representative photomicrographs. The concordance between the two independent observers was 100%, highlighting the robustness of the analysis.

**Table 2:** Summary of Immunohistochemistry results for E2F5 antibody tested on OEC

Type of tissue	Number of cases	Number positive	Percentage positive
Fimbrial/Paratubal/Paraovarian cyst	6	0	0

Surface epithelium/inclusion cysts	14	0	0

Endometriotic cyst	4	0	0

Serous cystadenoma	8	0	0

Mucinous cystadenoma	5	0	0

Benign stromal tumour	4	0	0

Dermoid tumour (mature)	12	0	0

Total	67	0	0

Early and late malignant cases			

Serous borderline and malignant E6; L8	24 (4+20)	11(2+9)	45.8

Mucinous borderline and malignant E11; L1	13 (6+7)	4(1+3)	30.77

Endometroid borderline and malignant E7; L5	16 (2+14)	8(1+7)	50

Clear cell carcinoma E2; L1	4	0	0

Adenocarcinoma (not further specified) E2; L2	4	2	50

Mixed adenocarcinoma E3; L1	7	6	85.7

Total	68	31	45.6

Note: Stage I/II (E); Stage III/IV (L) and data not available 5

#### Measurement of CA-125 level in serum and cyst fluid using sandwich ELISA method

The CA-125 levels were determined using a CA-125 ELISA kit (Alpha Diagnostic International, San Antonio, Texas, USA). The samples were diluted ten times using wash buffer and 25 μl of standards, control, and samples were pipetted into a 96-well plate in duplicate. Next, 100 μl of biotinylated anti-CA-125 capture antibody was loaded and the plate was incubated for 2 hours at room temperature. After washing, 100 μl of HRP conjugated anti-CA-125 antibody was added and the plate was incubated for 1 hour at room temperature followed by addition of substrate and the stop solution. The absorbance was read at 450 nm using the ELISA reader (Tecan, Seestrasse, Männedorf, Switzerland).

#### Western blotting

Once blood was collected it was allowed to clot. After centrifugation, the supernatant was collected and protein content was evaluated by the Bradford assay. To perform Western blot analysis for AREB6, ELF3, PAX8 and E2F5, 20 μg of protein from each sample was separated using 10% sodium dodecyl sulfate-polyacrylamide gel electrophoresis. Following electrophoresis proteins bands were transferred onto nitrocellulose membranes (Hybond-C Extra) (Amersham Biosciences UK Limited Buckinghamshire, UK). Filters were blocked in TBST containing 5% dried skimmed milk and 0.05% Tween-20 for 1 hr at room temperature. Thereafter, the filters were incubated over night at 4°C with primary antibodies diluted in TBST containing 5% skimmed milk and 0.05% Tween-20. Primary antibodies directed against the AREB6 (AVIVA Systems Biology, San Diego, California, USA), E2F5 (Acris GmbH, Hiddenhausen, Germany), PAX8 (Abcam Limited, Cambridge, UK), and ELF3 (Orbigen Inc, San Diego, CA) were chosen as the four TFs suggested by the bioinformatics analysis. Filters were washed 3 times 10 minutes each with TBST (0.05% Tween-20). They were subsequently incubated with HRP conjugated secondary antibody diluted 1:5000 in TBST containing 5% dried skimmed milk and 0.05% Tween-20 for 45 minutes at room temperature. Immunoreactivity was detected using the SuperSignal West Pico Chemiluminescent Substrate (Pierce biotechnology Inc, Rockford, USA) and once blotted the membranes were exposed to Pierce CLXPosure Film (Pierce biotechnology Inc, Rockford, USA).

### Statistical Analysis

The usefulness of E2F5, CA125, and their combinations as diagnostic markers to differentiate normal cases, benign, early and late malignant tumors were assessed using ROC analysis with sensitivity, specificity, positive & negative predictive values (PPV & NPV) presented. Analyses were performed using SPSS 15.0 (SPSS Inc., Wacker Drive, Chicago, Illinois) with statistical significance set at p < 0.05.

## Results

### Bioinformatics analysis

**Figure 1:**
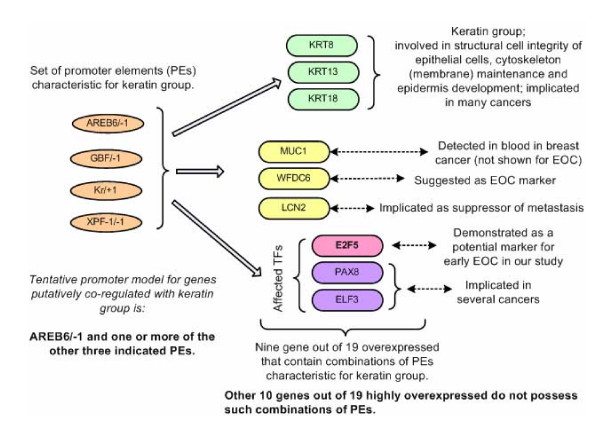
**Genes putatively controlled by tentative promoter model of keratin group**. Summary of the main finding in analysis of 19 genes over-expressed in ovarian cancer.

We identified TF encoding genes that were putatively controlled by PEs common to the keratin group (KRT8, KRT13, KRT18) (Figure 1), and are also previously reported to be at least 5-fold over-expressed in early and late stage ovarian cancer. Analysis suggests that these three keratin genes are controlled by AREB6/-1, and at least one of GBF/-1, Kr/+1, or XPG-11/-1 PEs. This keratin 'promoter model' is also shared with the three TF encoding genes (E2F5, PAX8, ELF3) and the other three genes (from the group of 19 analyzed). These other three genes are MUC1 (GeneID 4582, cell surface mucin glycoprotein, epithelial membrane antigen) which is elevated in the serum of patients with breast cancer [38]; WFDC6 (HE4) (GeneID 140870) a small serine proteinase inhibitor [39] that is part of a family thought to be a potential OEC marker [40] and LCN2 (GeneID 3934, lipocalin 2, oncogene 24p3) which has been shown to be an epithelial inducer in Ras malignancy and a suppressor of metastasis [41]. The outcome of our analysis suggested four TFs as potential diagnostic markers for OEC: E2F5, PAX8, ELF3 and AREB6. The logic of selecting these four TFs is as follows. Keratins are proteins involved in cellular integrity. Their abnormal expression may cause cell brake, thus allowing cell content to leak out and enter into body fluids [42-44]. Consequently, over-expressed TF encoding genes could result in overexpressed TFs that could thus enter blood and may be detectable there. This suggests that E2F5, PAX8 and ELF3 are candidates for this type of test as they are likely to be controlled in a similar fashion as the three keratin genes we considered and are also highly co-expressing with them in OEC conditions. AREB6 is the compulsory component of our putative 'keratin promoter model'. Although not over-expressed in OEC it could be the trigger of over-expression of keratins and the implicated three TFs. Thus we included it in our test list of potential markers.

### Tissue array studies

**Figure 2:**
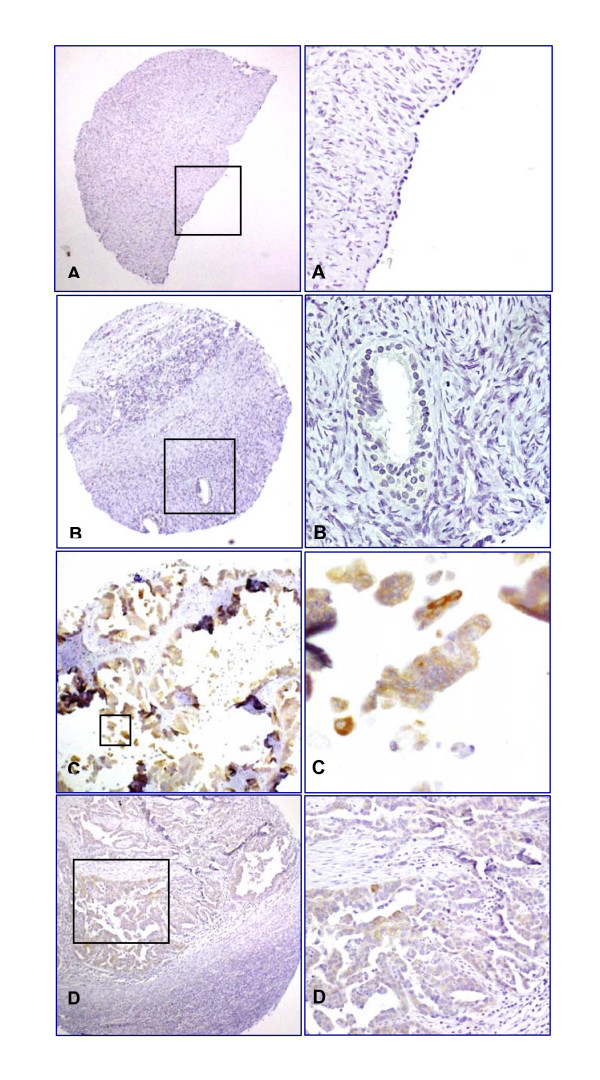
**Differential expression pattern of E2F5 in ovarian tumour specimens examined by immunohistochemistry**. A, benign serous cystadenoma, B, epithelial inclusion cyst, C, cystic serous carcinoma, and D, endometrioid adenocarcinoma samples. The low power photomicrograph in each section shows the full tissue microarray "punch" (x100), while the high power photomicrograph shows detail of the antibody expression (x600). There is no E2F5 expression in normal and benign tissue (A, B), but significant expression in ovarian epithelial cancer (C, D).

IHC on ovarian TMA constructs of normal ovarian epithelium, benign ovarian cysts, and early and late OECs showed that E2F5 expression was completely absent in all normal and benign samples tested (Figure 2; Table 2). Most of the E2F5 activity was localized in the cytoplasm with occasional nuclear membrane accentuation (See figure 2). In approximately half of all epithelial ovarian borderline and malignant tumours, which included serous, mucinous, endometroid and other subtypes, E2F5 expression was demonstrable by IHC, compared with none of the normal and benign cases (z = 6.1; p < 0.001; n = 135) (Table 2). The following expression pattern was observed for the different classes of OEC used for the current study (Table 2); Serous borderline and malignancy (11/24), Mucinous borderline and malignancy (4/13), Endometroid borderline and malignancy (8/16), Clear cell carcinoma (0/4); Adenocarcinoma NOS (2/4); and Mixed adenocarcinoma (6/7). Relatively high expression of E2F5 was found in endometroid and serous carcinoma than in other types of OEC. There was no difference in the IHC expression pattern observed between the right and left ovaries where tumour was bilateral.

### Detection of candidate proteins in serum using western blot

**Figure 3:**
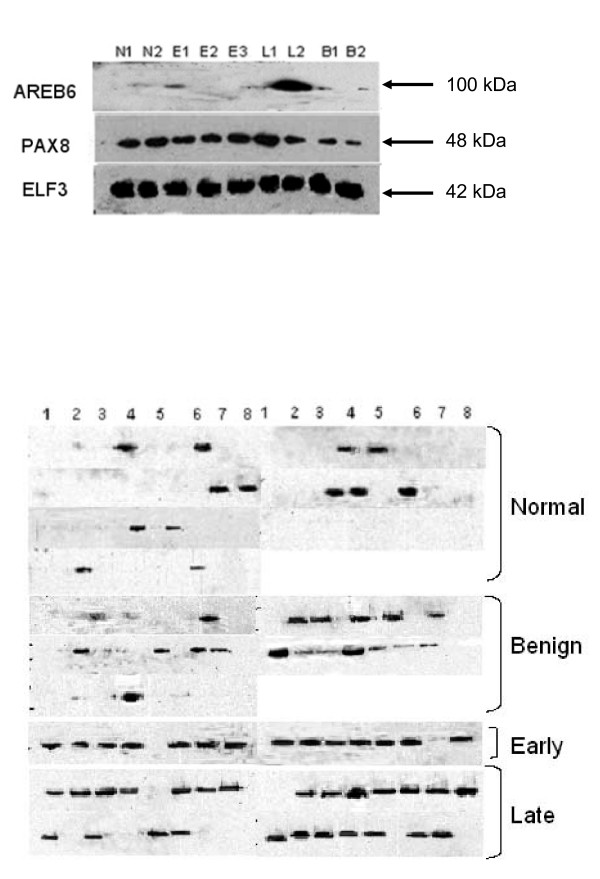
**Validation of potential target (AREB6, PAX8, ELF3) using western blots (A) and expression of E2F5 in serum from normal, benign and, malignant patient samples (B)**. (A) Target validation using western blots of AREB6, PAX8, ELF3, in serum of healthy volunteers (N) (n = 2), patients with benign ovarian cysts (B1, B2) (n = 2), and patients with late- (L1, L2) (n = 2) and early-stage (E1-E3) (n = 3) serous adenocarcinoma. AREB6 was overexpressed in late stage disease, but was not discriminatory for early cancer. PAX8 and ELF3 were equally expressed in all serum samples. (B) Expression of E2F5 in serum from normal (n = 56), benign (n = 40) and, malignant (n = 48) serum obtained from patients with cancer of the ovary.

Potential markers identified though bioinformatic approach were validated using western blotting approach. Transcription factors AREB6, PAX8, and ELF3 (Figure 3A) showed inconsistent patterns of expression when the study was carried on a limited number of samples. For TF AREB6, expression was found only in one malignant sample, while for PAX8 and ELF3 the expression pattern was present in normal and cancer samples. E2F5 expression was found to be prominent in cancer samples (early and late malignant cases) and not in normal serum samples. Hence, we carried out a comprehensive testing for E2F5 expression pattern on a total of 144 samples comprising of 56 normal; 40 benign, 16 early, and 32 late cases (Figure 3B). E2F5 expression (Table 3) was found in 23.21% (13 out of 56) healthy volunteers, but was present in 81.25% (39 out of 48: 16 early and 32 late cases; n = 48) of OEC patient serum. There was only 42.5% (17/40) expression of E2F5 in benign cases. Our data suggest that E2F5 could prove a highly discriminatory marker for detection of OEC, and in particular early OEC. Among the different subtypes of OEC, different expression pattern was observed between early and late types. In the early stage carcinoma, stage I/II endometroid (5/6); serous carcinoma (1/2); mucinous carcinoma (4/4); clear cell carcinoma (3/3) and others (1/1). In the stage III/IV cases of OEC cases the endometroid (8/9); serous carcinoma (15/19); mucinous carcinoma (1/1); clear cell carcinoma (1/3). Similar to tissue array results endometroid and serous carcinoma showed increased over expression of E2F5 compared to other types of OEC (cc = 0.47).

**Table 3:** Details of E2F5/CA125 expression pattern on different subtypes of OEC performed using western blotting technique.

	E2F5/CA125		E2F5/CA125		E2F5/CA125	
	Benign		Early		Late	
	Yes	No	Yes	No	Yes	No
Serous neoplasms	3/3	2/3	1/1	1/1	15/18	4/1
Mucinous neoplasms	4/1	7/10	4/3	0/1	1/1	0/0
Endometroid neoplasms	6/9	8/5	5/5	1/1	8/8	1/1
Clear cell neoplasms	0/0	0/0	3/2	0/1	1/1	2/2
Others	4/5	6/4	1/1	0/0	0/0	0/0

### CA125 assays

Only 75% of early cancer cases showed higher levels of CA125 compared to 87.5% for E2F5 expression in early OEC cases (Table 3). However, for late and benign cases CA125 performed better than E2F5. Statistical analysis showed no correlation between CA125 and E2F5 expression pattern (between different subtypes and between early and late in E2F5 and CA125).

### Statistical inference on CA125 and E2F5 expression in normal and malignant cases

Using data from 144 samples we checked the expression for both E2F5 and CA125 on serum from normal condition, benign cyst, malignant cyst (early and late conditions). We evaluated E2F5 either alone or in combination with CA125 for their efficacy in monitoring the malignancy status for OEC. We looked at the following conditions for their efficacy in diagnosing OEC and for accessing the sensitivity as well as specificity in diagnosing the malignancy associated with ovarian cancer: (1) CA125 used alone; (2) E2F5 used alone; (3) both E2F5 and CA125 negative; (4) at least one positive for either CA125 or E2F5; (5) at least one negative for either CA125 or E2F5; and (6) both positive for CA125 and E2F5,. A unique trend in the pattern of expression was observed for E2F5 along with CA125. Presence of CA125 or E2F5 in sera resulted in the increase in sensitivity, while a test positive for both CA125 and E2F5 led to a major increase in the specificity, PPV and NPV. This demonstrates that E2F5 is characteristic for a different subclass of individuals compared to CA125 which justifies utilization of E2F5 jointly with CA125.

From a clinical stand point, the key issue is to know the malignancy status of a cyst following a positive ultrasound scan for a cyst. To answer the question, after identification of a cyst using ultrasound, we used a combination of CA125 and E2F5. If both were absent, it could be considered as non-cancer case with 93.75% NPV, while the presence of either CA125 or E2F5 is capable of detecting OEC in 97.92% of the actual cancer cases (sensitivity). The presence of both CA125 and E2F5 makes the specificity as high as 72.50% as compared to CA125 alone (55%), E2F5 alone (55%) or "CA125 or E2F5" (37.5%). However, more samples have to be tested to assess real clinical utility of E2F5 as a biomarker for OEC.

The mixture of samples obtained from different ethnic populations prompted us to look into the association of race with the incidence of disease. The study showed that race did not have a role in the incidence of disease while considering expression pattern of both E2F5 and CA125. Similarly, while taking into account the age >40, as well as factors such as ethnicity and age >40, was also found to have no correlation with the occurrence of the disease. Our retrospective analysis therefore provides evidence that E2F5 could also be a useful independent prognostic indicator in patients with OEC as it targets different phenotypes compared to CA125. Moreover, the combined use of E2F5 and CA125 increases accuracy of OEC diagnosis significantly. Overall, our data suggest that presence of E2F5 in preoperative sera is associated with better survival outcome. More studies are, however, warranted to confirm these findings.

### Malignancy prediction

Using 38 benign and 48 malignant cases, we generated an additional artificial set of 602 cases using SMOTE algorithm. We trained the Kstar classification model on this artificial data set of 602 cases and then applied so trained model to the original 86 cases (48 malignant and 38 benign). The goal is to identify malignant cases. Our method was able to predict 47 out of 48 malignant cases as malignant, and had one benign case predicted wrongly as being malignant. This produced sensitivity of 97.92% (47/48), ppv of 97.92%, and specificity of 97.37% (37/38) (Table 4). At the same time, RMI > 200 criterion produces sensitivity of 77.08% (37/48) and specificity of 92.11% (35/38) (Table 5). Consequently, the F-measure and accuracy of the new method for assessing malignancy is increased from 84.08% and 83.72% based on RMI > 200 criterion up to 97.92% and 97.67%, respectively. Moreover, there is an increase of 20.84% of absolute scale in sensitivity with the new method, while simultaneously it increased specificity by 5.56% of absolute scale. Overall, this is a significant increase in accuracy. We evaluated the contribution of both CA125 biomarker and E2F5 status to the accuracy of separation of malignant from benign cases (Table 5). For that, we first excluded information about CA125 from the set of 13 features we considered. The sensitivity remained the same as with the all 13 features, but specificity was reduced to 94.74%. In another experiment we excluded from the original 13 features the E2F5 status. The sensitivity remained the same as when the original 13 feature are used, but specificity reduced to 92.11%. Thus, the addition of E2F5 to the set of features that we used reduces the false positive cases 3-fold (from 7.89% to 2.63%). These results demonstrated that only by the use of both CA125 and E2F5 information in combination with the other clinical features, one achieves the best classification performance. Thus the information about E2F5 status has proved to be beneficial for predicting malignancy.

**Table 4:** Malignancy prediction using Kstar classification as well as using RMI index for ovarian cancer.

	Kstar classification (Our method)	RMI > 200 criterion
Sensitivity	97.92% (47/48)	77.08%(37/48)
Specificity	97.37% (37/38)	92.11%(35/38)
PPV	97.92%	92.5%

**Table 5:** Diagnostic accuracy developed for ovarian cancer detection with 13 features using Kstar classification based on SMOTE algorithms.

	With 13 features	CA125 excluded from 13 features	E2F5 excluded from 13 features	RMI > 200
Sensitivity	97.92%	97.92%	97.92%	77.08%
Specificity	97.67%	94.74%	92.11%	92.11%

### Discussion

There is increasing evidence on the role of TFs as markers for cancer [24-26], as potential prognostic markers [45], and as targets for drug therapy [46]. Our assumption was that regulatory genes that are differentially over-expressed during the development and progression of OEC are likely to alter simultaneously the expression of many genes in the considered disease state, and that upregulation of gene clusters and an abnormal elevated expression of several unrelated genes could be explained if they shared similar promoter model, thus being potentially subjected to the similar regulatory mechanism. If the identified TFs are to have any utility as clinical markers, the ability to differentiate healthy controls and patients with benign conditions from patients with early-stage disease (as opposed to advanced-stage disease) is essential.

Regarding E2F5 we have unambiguously shown that it is characteristic for a group of patients different from those that have elevated CA125. This means that cases that will slip through CA125 test could be captured by E2F5 status. Second, we did show that E2F5 status has been remarkably specific and indicative on the tissue array tests. Thirdly, using E2F5 status in addition of other clinical indicators, enabled us to obtain extremely high accuracy in separation of malignant from benign cases that was not possible without E2F5.

Given the higher E2F5 levels in malignant tissues, approximately half of all malignant tissues showed expression for E2F5 while none of the normal and benign samples showed expression for E2F5. E2F5 belongs to the family of E2F TFs which are both proliferation promoting and proliferation inhibiting TFs, and E2F5 falls in the later category [47]. It is likely that elevated E2F5 levels might be attributed to its increased production during cancer as a means by the body to arrest the proliferation of tumour cells during the early stage of the disease. Our study on E2F5 in tissues and serum establishes the importance of E2F5 (a proliferation inhibiting gene), [48] as a potential marker for early OEC. Other studies also showed similar observations for this protein. E2F5 was found to be upregulated (5 fold) in early and late stage ovarian tumours [28]. Also, studies conducted using custom made microarray specific for OEC (Ovachip) showed upregulation of E2F5 in OEC [49], and it is found to play a key role in the neoplastic transformation of various cancer tissues as identified using microarray analysis [19]. Similar observations were recently highlighted by microarray experiments conducted on sporadic colorectal carcinoma tissues showed upregulation of E2F5 genes during malignancy [50,51]. The oncogenic property of E2F5 was discovered by the amplified E2F5 expression in primary rodent cells as well as in breast cancer tumours [52]. While in other cancers, a high molecular weight E2F complex is detected within human colon carcinoma cells when the cell-cycle is disrupted [53]. It was found that interferon-gamma treatment for ovarian cancer caused a reduction of the proliferation-promoting TFs E2F1 and E2F2, at the same time it also increased the inhibiting TFs E2F4 and E2F5[54]. This observation highlights the significance of E2F cross-talk in the anti-proliferative function of interferon-gamma [54].

E2F class of cell-cycle genes happen to be the key transcription factors involved in the transition from G to G1 phase have been recently the focus of attention for gynecological cancers [55]. E2F genes are a family of genes comprising 8 different genes identified till to-date [48]. G1-S-phase transition in normal cells requires phosphorylation of the retinoblastoma protein pRb and the related proteins pRb2/p130 and p107 by CDKs. This process results in the release of E2F transcription factors controlling various genes required for DNA synthesis and cell-cycle control. Role of cell-cycle genes [22] and imbalance in the regulation of genes promoting and inhibiting cellular proliferation and apoptosis has been implicated in the oncogenesis of OEC [56]. Alteration in genetic control in cancers is usually attributed to base pair mutations, but may also occur due to TF deregulation [55]. Deregulation of both proliferation-promoting and proliferation-inhibiting E2F TFs and their cross-talk were reported to influence the clinical outcome [55]. DNA amplifications for E2F5 (8p22-q21.3) has been observed during sporadic colorectal cancers [51]. The above evidence suggests that E2F5 is similar to MYC-type cooperating oncogene and persistent unregulated expression of E2F5 can assist other oncogenes to promote cell transformation. Along with chromosomal amplifications and overexpression of the E2F5 gene as detected in breast tumors suggests that E2F5 deregulation may have a role in human tumor development [52].

TFs have been described as markers for cancer as well as markers for prognostication [26,45,46]. TF Ets-1 has been found to have significant prognostic value for relapse-free survival as an independent predictor of poor prognosis in breast cancer. This observation correlates to its role in transcriptional regulation of factors involved in angiogenesis such as VEGF and extracellular matrix remodeling (PAI-1) [57]. Another TF, thyroid transcription factor 1 (TTF-1) has been found as a good prognostic factor for survival in non-small-cell lung cancer (NSCLC). Its effect appears also significant when the analysis is restricted to patients with adenocarcinoma [45]. Similarly microphthalmia TF has been reported as a marker for circulating tumor cell detection in blood of melanoma patients [26].

One can raise a question why it should be possible to look for TF in sera as biomarkers of ovarian cancer. Our methodology has been to look for those TFs that potentially affect genes producing proteins that influence extracellular matrix, or to those TFs that are both co-regulated and co-expressed with such genes. In our case we focused on keratins. We hypothesize that interplay of extracellular matrix and cancerous cells makes them more prone to braking so that cancer cell content leaks out and enters lymph and blood systems[42]. For this reason we expect to be able to find E2F5 in serum of patients with early and late stage OEC and our experimental results suggest that this may be the case.

Finally, one may argue that it was not necessary to do any bioinformatic analysis of gene expression data and directly test all over-expressed TFs. It is true that it was possible to make such tests directly, but our analysis enabled us focus on specific TFs, and also has provided a potential explanation for the co-expression of E2F5 and keratin genes in OEC based on the putative co-regulation mechanism suggested by our derived promoter model.

## Conclusion

Our findings provide additional support for the involvement of the E2F5 gene which belongs to the E2F family of genes in human OEC development and progression. While additional larger prospective studies are essential to validate our findings, we have demonstrated that the application of microarray analysis can facilitate the identification of genes, genetic pathways, and proteins that are not only involved in the pathogenesis of OEC but also may represent potential serum markers.

## Abbreviations

CA125: Cancer antigen 125; OEC: Ovarian Epithelial Cancer; FIGO; International Federation of Gynecology and Obstetrics; ORI: Over Representation Index; PE: Promoter Element; PPV: Positive Predictive Value; NPV: Negative Predictive Value; SMOTE: Synthetic Minority Oversampling T**e**chnique; TMA: Tissue Microarray; IHC: Immunohistochemistry; TF: Transcription Factor; TFBS: Transcription Factor Binding Site; TSS: Transcription Start Site

## Competing interests

The authors declare that they have no competing interests.

## Authors' contributions

Author 1 (NK), Author 2 (VBB), and Author 6 (MC), were involved in the concept, design and writing of the manuscript. Bioinformatics analysis and statistical analysis were carried out by Author 2 (VBB) and Author 4 (CYH). Author 6 (KR) was involved with sample procurement. Author 1 (NK), Author 3 (PNKB), Author 4 (PBK), and Author 5 (MST) carried out the validation studies including tissue microarray and western blotting studies. All authors read and approved the final manuscript.

## Pre-publication history

The pre-publication history for this paper can be accessed here:

http://www.biomedcentral.com/1471-2407/10/64/prepub

## Supplementary Material

Additional file 1 **Details of the modalities followed for analyzing over expressed genes in ovarian cancer**. (1) the details of the modalities performed for analyzing overexpressed genes in ovarian cancer (2) information on transcription factors AREB6 and PAX8 (3) a description on the potential utility of transcription factors in therapeutic applications and (4) relevant references for this section. Click here for file
