# Supplementary material for: E2F5 status significantly improves malignancy diagnosis of epithelial ovarian cancer
Source: BMC Cancer. 2010 Feb 24;10:64. doi: 10.1186/1471-2407-10-64 (PMC2841139; doi:10.1186/1471-2407-10-64)
Supplement: Additional file 1 — Details of the modalities followed for analyzing over expressed genes in ovarian cancer. (1) the details of the modalities performed for analyzing overexpressed genes in ovarian cancer (2) information on transcription factors AREB6 and PAX8 (3) a description on the potential utility of transcription factors in therapeutic applications and (4) relevant references for this section. [file 1471-2407-10-64-S1.PDF]

Additional files

E2F5 status significantly improves malignancy diagnosis of epithelial ovarian cancer

Narasimhan Kothandaraman[1]*, Vladimir B Bajic[2]*[§], Pang NK Brendan[3], Chan Y Huak[4], Peh B Keow[3], Khalil Razvi[5], Manuel Salto-Tellez[3] and Mahesh Choolani[1]*[§]

Details analysis of modalities followed for analysing overexpressed genes in ovarian cancer.

In defining commonality among different regulatory genes used for the current study, we took into consideration both the TFBS and the strand on which the TFBS is found. We further looked for other genes out of 19 highly over-expressed f that have a common promoter model characterized by the presence of AREB6/-1 and at least one of GBF/-1, Kr/+1, or XPG-11/-1, which is characteristic for keratin subgroup. We found the following genes have promoters that fit into this model: (a) MUC1 (cell surface mucin glycoprotein, epithelial membrane antigen) which can be found is serum in breast cancer cases [1]; (b) PAX8 which is involved in cancer growth, cell-differentiation and morphogenesis; (c) WFDC6 which is epidermal secretory protein (HE4) amplified in ovarian cancer and suggested as a potential ovarian cancer marker [2, 3]; (d) LCN2 whose role is suggested in epithelial differentiation and transport (LocusID 3934); and (e) E2F5 which is one of the key regulators of cell-cycle, also reported as an oncogene, [4], and shown to interact with tumor-suppressor proteins (LocusID 1875) [4-7]. Thus, we finally shortlisted the TFs within this group PAX8, ELF3 and E2F5 (Figure 1),

suggesting to us that the potential TFs were AREB6, PAX8, ELF3 and E2F5 which all were previously reported to play a key role in oncogenesis.

We further examined the expression pattern of E2F5, PAX8 and ELF3 in the original microarray expression data searching for consistency of expression and relative over-expression in early- versus late-stage disease. As anticipated from the microarray data and confirmed by these experiments, PAX8 and ELF3 were non-discriminatory in that they were present in all samples tested including those from healthy volunteers (Figure 3A). PAX8 was not consistently over-expressed in all ovarian cancer specimens, though it has been previously reported to be overexpressed in ovarian cancer [8-10] and ELF3 showed no stage-specific pattern in our samples. Analysis up to this point suggested that E2F5 was the most likely candidate TF that could prove a useful biomarker for detection of early OEC. To validate this possibility, we examined the E2F5 protein expression within ovarian tissue using ovarian TMA constructs, and studied its presence in serum of patients with OEC and controls. Cell-cycle genes are involved with frequent events in human cancers including ovarian cancer. Defects in many of the genes that regulate the cell-cycle have been implicated in cancer development and progression [5, 11]. The E2F family of genes has been targeted in tumorogenesis associated with ovarian cancer [5-7] and other cancers have shown deregulation of activity for E2F genes [4, 5, 12]. Therefore, we selected E2F5 gene primarily since it possessed, based on our bioinformatics analysis, a similar promoter model to those keratin genes that over-expressed in OEC.

Information on AREB6 and PAX8

The gene AREB6 has been found to be involved in cell division and spread of breast cancer especially during the late stage. Its activity has been found to increase and to be independent of estrogen contrary to its dependence on estrogen during the early stage of the disease [13]. Similarly, other genes identified in this study have also been implicated to play a key role on the neoplastic transformation of ovarian cancer. They include PAX8 which showed over expression in ovarian cancer [8, 9] and was shown to play a key for cancer survival for mostly all solid type cancers [14].

Potential utility of transcription factors in therapeutic applications.

Furthermore, the majority of oncogenic signaling pathways converge on sets of TFs that ultimately control gene expression patterns resulting in tumor formation and progression as well as metastasis. Since many of these TFs are inactive under normal physiological conditions and their expression and activities are tightly regulated, these TFs represent highly desirable and logical points of therapeutical interference in cancer development, progression and prognostication [15-17]. Detection of tumorigenic markers mostly proteins [18-20] and recently DNA [21, 22] and in serum and plasma has been investigated by different groups over the years. However lack of sensitivity for early disease and lack of specificity have been the major Achilles' heel for the diagnosis early OEC [20, 23]. The above findings suggest that blood is a rich source of tumour associated markers which are yet to be identified. It is likely that the changes in the expression profiles of regulatory genes within cancer tissue could be reflected, and be

detectable, in the patients' circulating blood, potentially allowing for early diagnosis, tailored therapy, and monitoring of disease.

References

[1] Croce, M. V., Isla-Larrain, M. T., Demichelis, S. O., Gori, J. R., *et al.*, Tissue and serum MUC1 mucin detection in breast cancer patients. *Breast Cancer Res Treat* 2003, *81*, 195-207.

[2] Hellstrom, I., Hellstrom, K. E., SMRP and HE4 as biomarkers for ovarian carcinoma when used alone and in combination with CA125 and/or each other. *Adv Exp Med Biol* 2008, *622*, 15-21.

[3] Hellstrom, I., Raycraft, J., Hayden-Ledbetter, M., Ledbetter, J. A., *et al.*, The HE4 (WFDC2) protein is a biomarker for ovarian carcinoma. *Cancer Res* 2003, *63*, 3695-3700.

[4] Polanowska, J., Le Cam, L., Orsetti, B., Valles, H., *et al.*, Human E2F5 gene is oncogenic in primary rodent cells and is amplified in human breast tumors. *Genes Chromosomes Cancer* 2000, *28*, 126-130.

[5] Chen, C., Wells, A. D., Comparative analysis of E2F family member oncogenic activity. *PLoS ONE* 2007, *2*, e912.

[6] Reimer, D., Sadr, S., Wiedemair, A., Goebel, G., *et al.*, Expression of the E2F family of transcription factors and its clinical relevance in ovarian cancer. *Ann N Y Acad Sci* 2006, *1091*, 270-281.

[7] Reimer, D., Sadr, S., Wiedemair, A., Stadlmann, S., *et al.*, Clinical relevance of E2F family members in ovarian cancer--an evaluation in a training set of 77 patients. *Clin Cancer Res* 2007, *13*, 144-151.

[8] Schaner, M. E., Ross, D. T., Ciaravino, G., Sorlie, T., *et al.*, Gene expression patterns in ovarian carcinomas. *Mol Biol Cell* 2003, *14*, 4376-4386.

[9] Hibbs, K., Skubitz, K. M., Pambuccian, S. E., Casey, R. C., *et al.*, Differential gene expression in ovarian carcinoma: identification of potential biomarkers. *Am J Pathol* 2004, *165*, 397-414.

[10] Bowen, N. J., Logani, S., Dickerson, E. B., Kapa, L. B., *et al.*, Emerging roles for PAX8 in ovarian cancer and endosalpingeal development. *Gynecol Oncol* 2007, *104*, 331-337.

[11] Milde-Langosch, K., Riethdorf, S., Role of cell-cycle regulatory proteins in gynecological cancer. *J Cell Physiol* 2003, *196*, 224-244.

[12] Rhodes, D. R., Yu, J., Shanker, K., Deshpande, N., *et al.*, Large-scale meta-analysis of cancer microarray data identifies common transcriptional profiles of neoplastic transformation and progression. *Proc Natl Acad Sci U S A* 2004, *101*, 9309-9314.

[13] Bynthia M. Anose ; Sanders, M. M., Role of AREB6/ZEB Transcription Factor in Invasive Breast Cancer; Accession Number : ADA416730;. 2003.

[14] Muratovska, A., Zhou, C., He, S., Goodyer, P., Eccles, M. R., Paired-Box genes are frequently expressed in cancer and often required for cancer cell survival. *Oncogene* 2003, *22*, 7989-7997.

[15] Libermann, T. A., Zerbini, L. F., Targeting transcription factors for cancer gene therapy. *Curr Gene Ther* 2006, *6*, 17-33.

[16] Turner, D. P., Watson, D. K., ETS transcription factors: oncogenes and tumor suppressor genes as therapeutic targets for prostate cancer. *Expert Rev Anticancer Ther* 2008, *8*, 33-42.

[17] Crijns, A. P., Fehrmann, R. S., de Jong, S., Gerbens, F., *et al.*, Survival-related profile, pathways, and transcription factors in ovarian cancer. *PLoS Med* 2009, *6*, e24.

[18] Zhang, Z., Bast, R. C., Jr., Yu, Y., Li, J., *et al.*, Three biomarkers identified from serum proteomic analysis for the detection of early stage ovarian cancer. *Cancer Res* 2004, *64*, 5882-5890.

[19] Mor, G., Visintin, I., Lai, Y., Zhao, H., *et al.*, Serum protein markers for early detection of ovarian cancer. *Proc Natl Acad Sci U S A* 2005, *102*, 7677-7682.

[20] Bast, R. C., Jr., Badgwell, D., Lu, Z., Marquez, R., *et al.*, New tumor markers: CA125 and beyond. *Int J Gynecol Cancer* 2005, *15 Suppl 3*, 274-281.

[21] Chen, X., Bonnefoi, H., Diebold-Berger, S., Lyautey, J., *et al.*, Detecting tumor-related alterations in plasma or serum DNA of patients diagnosed with breast cancer. *Clin Cancer Res* 1999, *5*, 2297-2303.

[22] Kopreski, M. S., Benko, F. A., Gocke, C. D., Circulating RNA as a tumor marker: detection of 5T4 mRNA in breast and lung cancer patient serum. *Ann N Y Acad Sci* 2001, *945*, 172-178.

[23] Bast, R. C., Jr., Brewer, M., Zou, C., Hernandez, M. A., *et al.*, Prevention and early detection of ovarian cancer: mission impossible? *Recent Results Cancer Res* 2007, *174*, 91-100.
